# Price and Affordability of Heart Failure Guideline Directed Medical Therapy in Venezuela: A Cross Sectional Observational Study

**DOI:** 10.5334/gh.1474

**Published:** 2025-10-09

**Authors:** Karim J. Gebran-Chedid, Diana De Oliveira-Gomes, Gabriela Lombardo, Maria Carolina Bacci-Padron, David A. Forero-Peña

**Affiliations:** 1 Department of Internal Medicine, New York Medical College/Metropolitan Hospital Center, New York, NY, USA; 2 Biomedical Research and Therapeutic Vaccines Institute, Ciudad Bolívar, Venezuela; 3 “Luis Razetti” School of Medicine (Alumni), Universidad Central de Venezuela, Caracas, Venezuela; 4 Department of Internal Medicine, UT Southwestern Medical Center, Dallas, TX, USA

**Keywords:** heart failure, guideline directed medical therapy, medication affordability, Venezuela

## Abstract

**Background:**

Heart failure (HF) impacts over 56 million people worldwide, with significantly higher mortality rates in low and low-middle-income countries (LIC/LMICs). Despite the effectiveness of guideline-directed medical therapy (GDMT) for HF with reduced ejection fraction (HFrEF), its use remains limited in LIC/LMICs due to limited availability and affordability. These barriers are particularly pressing in Venezuela’s context, as the country faces an ongoing crisis.

**Objective:**

Describe price and affordability of HF Guideline Directed Medical Therapy at optimal dosages in Venezuela.

**Methods:**

We conducted a cross-sectional analysis from December 2023 to January 2024, surveying prices of HF GDMT medications across 13 major pharmacy networks in Venezuela. Medications analyzed included ACE inhibitors (ACEi), angiotensin receptor blockers (ARB), beta-blockers (BB), mineralocorticoid receptor antagonists (MRA), angiotensin receptor-neprilysin inhibitors (ARNI), and sodium-glucose co-transporter 2 inhibitors (SGLT2i). Affordability was defined and calculated using the World Health Organization/Health Action International (WHO/HAI) methodology, comparing the median costs of one month of HF GDMT at optimal dosages to the lowest-paid government worker’s (LPGW) daily wages. Other comparisons of price affordability were made against the mean daily salary of managers, professional and non-professional workers in the country.

**Results:**

The most expensive medication regime for HF in Venezuela was ARNI-based GDMT with a median monthly cost of 393.81USD, followed by ARB-based GDMT and ACEi-based GDMT costing $100.88USD and $82.23USD respectively. meaning LPGW and elderly receiving retirement stipends would need between 506 to 2421 paid work days to cover one month of treatment at optimal dosages.

**Conclusion:**

Based on the WHO/HAI methodology all HF GDMT regimens were deemed unaffordable in Venezuela. Similar affordability challenges exist in other LIC/LMICs countries highlighting the need for global advocacy and policy action to address financial barriers to access guideline-based heart failure care.

## Introduction

Heart failure (HF) affects approximately 56.2 million people worldwide (1); low and low-middle-income countries (LIC/LMIC) face a one-year mortality rate 22% to 58% higher compared to high-income countries (2). Patients with HF in these countries are typically younger, often lack insurance, and are diagnosed at advanced disease stages (3). In the past decade, there have been significant efforts to decrease mortality in patients with HF, from educational initiatives aiming to broaden the availability of cardiovascular services training professionals in these regions to development of dedicated heart failure clinics, however, the outcomes remain worse in LIC and LMICs (4,5,6).

The treatment for HF with reduced ejection fraction (HFrEF), called guideline-directed medical therapy (GDMT), consists of: (1) inhibition of the renin-angiotensin-aldosterone system with either angiotensin-converting enzyme inhibitors (ACEi), angiotensin receptor blockers (ARB) or angiotensin receptor-neprilysin inhibitors (ARNI), (2) β-blockers (BBs), (3) mineralocorticoid receptor antagonists (MRA), and (4) sodium-glucose cotransporter 2 inhibitors (SGLT2i) (2). GDMT has been demonstrated to substantially reduce mortality and improve outcomes (Quality of life, hospital readmission rates, biomarker levels) in patients with HFrEF (1,7,8); however, despite compelling evidence supporting their effectiveness, it remains notably underutilized in LIC/LMICs (2). One of the main reasons being limited availability and affordability (1,9), as a result, there is an increased risk of major adverse cardiovascular events among people living in these regions (2,4).

Venezuela is in the midst of a severe economic and political crisis with escalating poverty rates and shortages of basic needs and medicines (10). The burden of cardiovascular disease in Venezuela accounted for 173.9 deaths and 3,400.4 Disability Adjusted Years Lost per 100,000 people in 2019 (11), with an estimated age standardized prevalence of HF ranging from 700 to <800 per 100,000 people in 2021 (12). For the country, the financial cost of HF including health system costs and productivity losses has been estimated at 522 million United States Dollar (USD) in 2017 (13). For the patients, mortality at one of the mains hospital in Caracas, was as high as 32.2% at 90 days following hospital discharge for acute decompensated HF (14). Current initiatives to reduce the burden of disease focus on prevention of risk factors like hypertension but either lack enough funding, are limited in location, or in very early stages of development as the case of Proyecto Hipertension Venezuela in partnership with World Health Organization/Pan American Health Organization (WHO/PAHO) HEARTS initiative (15).

One of the main limitations of studying HF in Latin America is the lack of consistent studies demonstrating treatment availability and outcomes (16). An important step to improve our understanding of the morbimortality and change the outcomes of patients with HF in Venezuela is to analyze the price, accessibility, and affordability of HF GDMT, which remains unmeasured in the country. This study aims to describe the current medication prices of HF GDMT at Optimal Medical Therapy (OMT) dosages and compare them with current living wages as a measure of affordability.

## Methods

### Study design

This is an observational cross-sectional study. We assessed the price and affordability of HF GDMT in Venezuela, from December of 2023 to January 2024 and aimed to follow the World Health Organization/Health Action International (WHO/HAI) methodology to evaluate medication prices and affordability (17). However, due to the lack of publicly available data we included only the private sector for analysis and did not include governmental and non-governmental organizations. We queried thirteen (13) retail pharmacy networks in the country with an estimated combined number of 728 stores distributed across the country as depicted in Supplemental 1.

### Pharmacies and medication selection

Pharmacies were selected from the ‘Cámara Venezolana de Farmacias’ (CAVEFAR) (18), an umbrella organization of pharmacy establishments, duly registered with the ‘Ministerio del Poder Popular para la Salud’ (MPPS). The sampling was non-random, selecting the largest pharmacy networks. All included pharmacies were retails with Business to Costumer service model (B2C). Wholesale pharmacies represent a Business to Business (B2B) model and since they don’t sell to individuals directly were excluded.

Additionally, authors agreed on adding two additional large pharmacy networks: the Unified System of Pharmaceutical Care (SUAF), with its government-supported mobile pharmacy business model, and ‘RedVital’ pharmacy, a network with a ‘wholesale pricing for the consumer’ model.

The medications queried were selected from the Section 7.3.8 of the 2022 AHA/ACC/HFSA guidelines recommendations for management of HF (8). Authors selected up to three medications from each pharmacological group with exclusion of active ingredients unavailable in the country (eg. Trandolapril, Fosinopril). Included medications are depicted in Table 1.

**Table 1:** List of medications included for analysis by group, presentation and targeted doses used for analysis.


MEDICATION GROUP	MEDICATION	PRESENTATION INCLUDED FOR ANALYSIS	2022 AHA/ACC/HFSA GUIDELINE DEFINED OPTIMAL MEDICAL THERAPY DOSE (8)	OPTIMAL MEDICAL THERAPY DOSE SELECTED FOR THE STUDY

MRA	Spironolactone	25 mg oral	25–50 mg once daily	25 mg once daily

	Eplerenone	25 mg oral	50 mg once daily	50 mg once daily

SGLT2 inhibitors	Dapagliflozine	10 mg oral	10 mg once daily	10 mg once daily

	Empagliflozine	10 mg oral	10 mg once daily	10 mg once daily

Beta Blockers	Metoprolol	95 mg oral*	200 mg once daily	190 mg daily*

	Carvedilol	12.5 mg oral	25–50 mg twice daily	25 mg twice a day

	Bisoprolol	5 mg oral	10 mg once daily	10 mg a day

ARNI	Sacubitril/Valsartan	24.3/25.7 mg oral	97 mg sacubitril and 10.3 mg valsartan twice daily	97.2 mg sacubitril and 102.8 mg valsartan twice a day**

ACEI	Enalapril	10 mg oral	10–20 mg twice a day	10 mg twice a day

	Ramipril	10 mg oral	10 mg once daily	10 mg once daily

	Lisinopril	20 mg oral	20–40 mg once daily	Lisinopril 40 mg once daily

ARB	Valsartan	80 mg oral	160 mg twice daily	160 mg twice daily

	Losartan	50 mg oral	50–150 mg once daily	150 mg once daily

	Candesartan	8 mg oral	32 mg once daily	32 mg once daily


Abbreviations: ACEI: Angiotensin Converting Enzyme Inhibitors; ARB: Angiotensin Receptor Blocker; ARNI: Angiotensin Receptor – Neprilysin Inhibitor; BB: Beta blocker; MRA: Mineralocorticoid receptor antagonist; SGLT2i: Sodium-Glucose Co-Transporter 2 inhibitors. * Metoprolol presentation available across Venezuela was 95 mg per tablet. ** Sacubitril/Valsartan presentation available across Venezuela was 24.3/25.7 mg.

### Definition of Optimal Medical Therapy

The definition of OMT for each medication group (ARNIs, ACEI, ARBs, BB, MRA, SGLT2i) is based on target treatment doses depicted in Section 7.3.8 ‘GDMT Dosing: Sequencing and Uptritation’from the 2022 AHA/ACC/HFSA guideline recommendations for management of HF (8) (Table 1).

### Data collection and analysis

We developed a structured form to collect retail prices for ACEi, ARB, BBs, MRA, ARNI, and SGLT2i from 13 of the most important retail pharmacies in the country. The search was conducted from December 2023 to January 2024. Searching methods included direct phone call to costumer services and web catalog searches.

All medications were queried across each of the selected pharmacy networks based on the active ingredient. When multiple brand-names or generic medications were available, we selected the cheapest alternative.

The target treatment cost was calculated based on OMT as per AHA/ACC/HFSA Guidelines (8) (Table 1), for this purpose, the three medication combination regimes recommended by the AHA/ACC/HFSA Guidelines for management of HF were selected for analysis: Group A (ARNI, SGLT2i, BB, and MRA), Group B (ACEI, SGLT2i, BB, and MRA) and Group C (ARB, SGLT2i, BB, and MRA) (8). To account for differences in the number of pills between the presentations, all medications underwent adjustment by calculating the price per pill, subsequently the number of pills required to attend the OMT each month were multiplied by the price per pill. To account for possible regional price variabilities across same-network pharmacies, prices of each medication were verified at up to five different locations per pharmacy network across the country to adjust for price differences when needed. Medication prices were calculated as numbers with means, standard deviations and median. Data was summarized using descriptive statistics, including mean, standard deviation (SD), median, frequency, and percentage (%). Statistical analyses were performed using the Statistical Package for the Social Sciences (SPSS) version 26 (IBM Corporation, Armonk, NY, USA).

### Affordability measurement

The WHO/HAI’s methodology in Medicine Prices, Availability, and Affordability project was used as a guideline to measure affordability (17), according to which affordability is measured as the required number of days for the wages of the lowest-paid unskilled government worker (LPGW) required to cost a treatment course, using the ratio monthly median out-of-pocket price/daily LPGW wage for every individual medication and regime combination at OMT included in the study. Medications whose monthly cost surpassed the LPGW’s daily wage –Affordably ratio (AR) >1- were labelled as non-affordable. Because the official minimum wage of LPGW in Venezuela in 2023 was 130 Bolivars per month, i.e., ~3.6, USD (exchange rate of Venezuela’s Central Bank), and to obtain a more accurate representation of Venezuelans’ income, we included a stratified assessment of affordability based on other incomes from data retrieved by Observatorio Venezolano de Finanzas (OVF) and stratified per position: Managers, professionals and non-professional workers (19). The AR for managers, professionals, and non-professional workers was also calculated with an adjustment of the formula to their salary: Monthly median Out-of-pocket price/Daily-Income.

## Results

The retail prices of each of the GDMT medications are listed in Table 2. The most expensive medication group from the list are ARNI’s with an OMT cheapest alternative across pharmacies mean price 321.04 ± 33.43 USD followed by SGLT2i’s pricing at 37.28 ± 17.80 USD and the cheapest group were MRA’s with a retail price of 12.31 USD. The most expensive GDMT regime is the ARNI-based combination (Group A) with a pharmacy retail mean price of 397.87 USD and the cheapest is the ACEI-based combination (Group B) with a retail mean price of 92.68 USD.

**Table 2:** Cost of each GDMT and Affordability Ratio stratified by salary.


MEDICINE GROUP	PRICE ($)				AFFORDABILITY RATIO			
	
	MEDIAN	MEAN +/– SD	MIN	MAX	MANAGERS*	PROFESSIONALS#	NON-PROFESSIONAL WORKERS ^	RETIREMENT/MINIMUM WAGE Δ

BB	21.34	27.24 ± 18.77	1.28	79.92	1.49	2.26	3.19	168.74

ARNI	319.81	321.04 ± 33.43	287.02	361.09	17.48	26.52	37.47	1978.34

ACEI	8.23	15.85 ± 19.49	0.13	63.12	0.86	1.30	1.84	97.4

ARB	26.88	20.48 ± 11.92	2.18	43.97	1.25	1.90	2.68	141.95

MRA	11.33	12.31 ± 7.54	2.93	23.29	0.66	1.01	1.43	75.64

SGLT2i	41.33	37.28 ± 17.80	11.9	63	2.02	3.07	4.33	229.09

**Group A:** ARNI + BB + MRA + SGLT2i	393.81	398.99 ± 89.84	303.13	527.30	21.39	32.44	45.84	2420.06

**Group B:** ACEI + BB + MRA + SGLT2i	82.23	92.91 ± 19.44	16.24	229.33	4.46	6.77	9.57	505.32

**Group C:** ARB + BB + MRA + SGLT2i	100.88	97.53 ± 16.14	18.29	210.18	5.47	8.31	11.74	619.93


Abbreviations: ACEI: Angiotensin Converting Enzyme Inhibitors; ARB: Angiotensin Receptor Blocker; ARNI: Angiotensin Receptor – Neprilysin Inhibitor; BB: Beta blocker; MRA: Mineralocorticoid receptor antagonist; SGLT2i: Sodium-Glucose Co-Transporter 2 inhibitors. Type of employment mean monthly salary in United States Dollar ($): * = 405; # = 267; ^ = 189; Δ = 3.6. Type of employment mean daily wages in United States Dollar ($): * = 18.4; # = 12.3; ^ = 8.59; Δ = 0.16.

**Figure 1:**
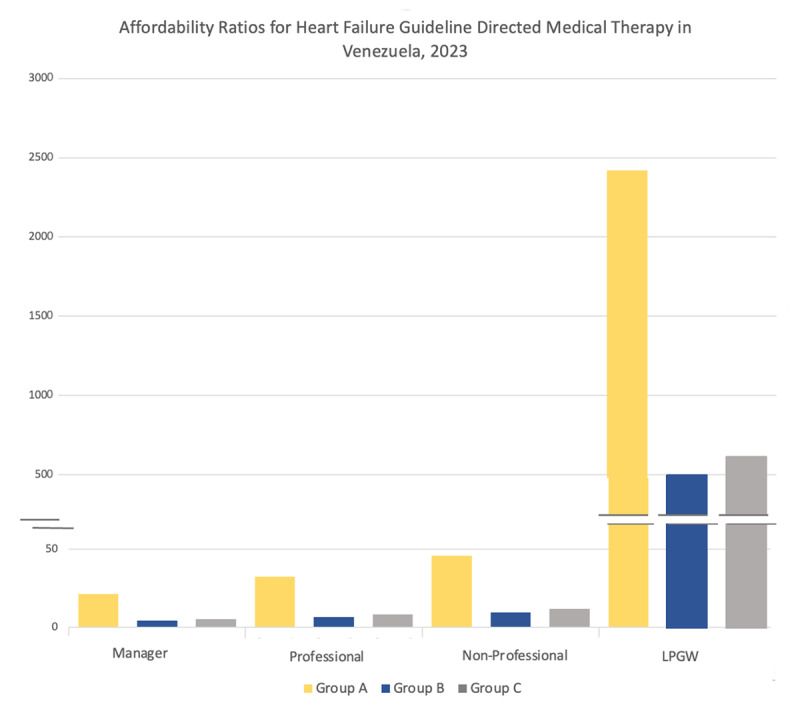
Affordability ratios for Heart Failure Guideline Directed Medical Therapy. Y axis displays affordability ratio, X axis displays income type. Each combination of Guideline Directed Medical Therapy for Heart Failure is designated as Group A (ARNI, SGLT2i, BB, and MRA), Group B (ACEI, SGLT2i, BB, and MRA) and Group C (ARB, SGLT2i, BB, and MRA) according to signalized color. Segmented line represents the affordability ratio cut-off defined by the WHO/HAI.

The AR for each medication, individually and in combination, is stratified by income type in Table 2. All included medications at target OMT were deemed unaffordable based on the WHO/HAI standard of comparison with LPGW, having an AR > 1. ARNI’s had the highest AR (1965.31), followed by SGLT2i (253.98), ARB (165.18), BB (131.13), MRA (69.62) and ACEI (50.57). Measurement of affordability adjusted to the income of managers, professionals, and non-professional workers also resulted in most medications being labeled as unaffordable with an AR > 1, except for MRA’s and ACEI’s which were affordable for managers (Figure 1, Table 2).

HF GDMT combination regimes at OMT were all deemed unaffordable, with Group A being the most unaffordable (AR of 2420.06), followed by Group C (AR 619.93) and Group B (AR 505.32). Measurement of affordability adjusted to the income of managers, professionals and non-professional workers also resulted in combinations being labeled as unaffordable (Table 2).

## Discussion

Our study shows that existing HF medication regimes at OMT in Venezuela are unaffordable. The existing AR is an important financial barrier for patients to obtain their medications and puts them at risk of higher all-cause mortality either due to financial toxicity or due to discontinuation (20,21). ARNIs were the most expensive medications in our study. The median price for one month of supply at OMT is 319.81 USD equivalent to 1979 work-days for the LPGW. In contrast, ACEIs were the least expensive at a median price of 8.23 USD. As expected, the most expensive medication regime was the ARNI-based GDMT with a monthly cost of 393.81USD, whereas the ACEI-based regime is much cheaper at 82.23 USD.

The scale of these findings reveals an urgent need to support Venezuelans living with heart failure overcome the severe financial barriers to treatment, especially in the context of the ongoing socioeconomic crisis. Beyond the national scope, our results draw attention to similar challenges in other LIC/LMIC within the region or countries facing political and/or economic instability. Collectively, our findings and those of researchers around the globe underscore the need to advocate for policies aimed at relieving the financial barriers for HF treatment.

### International context

Globally, the gap in utilization of GDMT for HF is poor. Results from a systematic review and metanalysis of 334 observational studies encompassing 1,5 million patients with HF from different geographic areas revealed a utilization of ~80% for BB, 82% for ACEIs/ARBs/ARNIs and 41% for MRA. Additionally, the study revealed significant disparities in utilization of GDMT between HIC and LMICs most of which can be attributed to economic factors (22).

The lack of adequate financial support is a major challenge for people seeking healthcare in LIC/LMICs. On average, 50% of health care funding in LICs comes from out-of-pocket expenses, compared to 30% in middle-income countries and only 14% in high-income countries (23). Additionally, only 38% of health care financing in LICs comes from pooled sources, such as government spending, public health insurance, and prepaid private insurance, which share the financial risks across the population (23). This contrasts with middle-income countries and high-income countries, where approximately 60% and 80% of health care financing is pooled, respectively (23). Moreover, the poorer the country, the greater the proportion of total health expenditure that is out-of-pocket (24) and high out-of-pocket expenses deter lower-income individuals from accessing necessary services, leading to untreated health issues (25).

In a survey of 10 LIC/LMICs it was noticeable the scarcity of newer medications such as ARNI and SGLT2i outside of the private sector, mainly due to lack of coverage under publicly funded programs (1). The results of the survey -in alignment with our findings- recall ARNIs (Sacubitril-Valsartan) as the most expensive drug among surveyed countries with a mean 30-day price ranging from 11.06 USD in Pakistan to 611.50 USD in the US, and the cheapest drug was the MRA spironolactone, with a mean 30-day price ranging from 0.18 USD in Pakistan to 12.32 USD in England. HF registry from 50 hospitals in Kerala, India, shows that approximately only 25% of patients received GDMT for HF; the study reveals a significant gap in GDMT prescriptions. Contributing factors include overcrowding in clinical settings and inadequate insurance coverage leading to high out-of-pocket costs for medicines, as observed in our study. Moreover, the ARNIs use was extremely low at just 2%, being affordability the main factor, as their cost is more than ten times that of ACE inhibitors and angiotensin receptor blockers, leading to higher discontinuation rates (26). A cross-sectional analysis of 53 LICs/LMICs on HF drug found that generic drugs were more widely available than originator brand drugs, especially in the private sector, with generic furosemide being the more affordable option (2).

### Local context

More than 7 million Venezuelan’s left the country as a result of the humanitarian crisis (27), and with them more than 24,000 physicians (28). The ‘brain drain’ in Venezuela was followed by the shattering of the healthcare system structure. By 2023, Venezuelan hospitals faced intermittent electricity and water supplies disruptions as well as shortages of laboratory services and medicines (29).

To outlive the crisis, Venezuelan’s remaining in the country received support through money remittances from relatives who left the country (30) or sought supplemental sources of income in foreign currency through online gigs (31), or the informal market. These alternative sources of income could potentially allow some Venezuelans to afford some of their HF medicines.

Data from Estudio Venezolano de Salud Cardio-Metabolica (EVESCAM) collected between 2014 and 2017 showed that approximately 67.4% of Venezuelans obtained their care at public health centers while 21% opted for private facilities either using their insurance (12,5%) or paying out of pocket (8,5%) (32). This numbers remained similar by 2022 with results from *Encuesta de Condiciones de Vida* (ENCOVI) 2022 -one of the main surveys of living conditions in the country- showing that 70% of surveyed participants sought their care at public facilities, while 20% did through private services and 7% at pharmacies (33). The 1999 Venezuelan constitution guarantees free healthcare for all citizens – including medications – yet there is no public data from official sources on the availability of medicines in public institutions, which obscures the dramatic situation of medication availability and affordability in Venezuela. Despite most Venezuelans receiving their care at public institutions the results of ENCOVI in 2023 revealed that 67% of Venezuelans had to pay out of pocket the full cost of all their prescriptions, while 14% had to pay for part of their prescribed medicines -some provided for free by the government, others paid in the private sector out of pocket- and 6% were unable to obtain any (34).

In both public and private sectors of the Venezuelan health system, when patients receive prescriptions for medicines they can use the recipe at any pharmacy of their preference either public or private to have their medications dispense, when public pharmacies are in a shortage or lack availability, they find themselves in the need to pay out of their own pocket at private retailers. For insured patients this is no different, as they pay upfront for their medications and only then can submit a claim to their insurance company for reimbursement, but due to the country’s rapid inflation and currency devaluation, the delayed reimbursement often in local currency leaves them at a financial disadvantage.

In 2022, 47% of Venezuelans included in ENCOVI 22 self-reported being insured by governmental entities, 20% through private insurance paid by a governmental entity, and 15% had insurance paid by private institution or themselves (33).

Venezuelans with HF are not the only ones facing barriers to accessing their medication in Venezuela; patients with other chronic illnesses such as diabetes and cancer also struggle to access necessary treatments due to basic medication shortages, in this order of ideas, by 2017 the estimated supply of basic medications barely reached a 15% (35). Infections are also on the rise, the political and economic crisis led to an uncontrolled spread of communicable diseases at alarming rates, including HIV, tuberculosis, and malaria, as well as the re-emergence of measles, pertussis, and diphtheria (10). The support of WHO/PAHO funds designated towards Plan Maestro in collaboration with Venezuela’s government has been a key reliever for citizens living with HIV or dealing with tuberculosis or malaria (36), yet, much is to be done to assist patients with disabling non-communicable diseases like HF. Initiatives like HEARTS Americas are a promising first step, by including in their plan access to essential medications such as ACEIs, ARBs, and Beta-blockers, which are key for patients at risk and living with HF (37), unfortunately, this program leaves out two game-changing pharmacologic groups: SGLT2i and ARNIs, which are the ones who carry the highest financial burden for patients living with HF in Venezuela.

### Future directions

Regional problems may benefit from regional solutions. In the past, intergovernmental collaboration between Latin American countries through MERCOSUR have shown success in reducing price of HIV and cancer medications (38). Attempting collective bargaining agreements with pharmaceutical companies for more affordable pricing of Sacubitril-Valsartan, Dapagliflozine and Empagliflozine -main drivers of current prices- could lower the burden on citizens across the Latin American. On the other hand, the existing HEARTS project from the PAHO could help relieve the existing financial burden incorporating Sacubitril-Valsartan and SGLT2 inhibitors to their medication technical package repository (37).

Local strategies to improve affordability of GDMT within Venezuelan border’s are also possible. First, strengthening local production through incentives such as tax breaks, subsidies or low interest loans. Second, considering compulsory licensing to local pharmaceuticals to produce essential GDMT medications could potentially reduce costs of patented medications (39). Finally, fostering community organization and reducing restrictions on non-governmental organizations (40).

### Limitations

The results of our study must be interpreted with caution and an understanding of the country’s ever-changing economic, political and social environment. Among the limitations of our study: First, we retrieved our wages from (OVF) whose report is based on an aleatoric sample from main urban cities located at the northwestern region of the country and may not depict the reality lived by citizens in rural areas or in the southeastern region of the country. Second, the pharmacies surveyed have different market shares and distribution across the country which are not being taken into account in this study. Finally, this study assesses the out-of-pocket cost of GDMT at OMT without taking in consideration insurance coverages, governmental and non-governmental organizations, which to some degree may ameliorate this weight but are inconsistent across the country.

## Conclusion

Our study on the price and affordability of HF GDMT in Venezuela demonstrates that current treatment regimens are unaffordable at optimal medical dosage. The magnitude of our findings underscores an urgent need to implement and advocate for initiatives to assist them and other patients living with HF in LIC/LMICs. Moreover, there is a pressing need to generate epidemiological and generalizable data on HF in Venezuela, expand access to and use of GDMT, invest in advanced HF therapies, and establish national HF registries—critical steps that would inform and strengthen future efforts to improve HF care.

**Supplemental 1:** Distribution of pharmacy retail stores by state and pharmacy network.


	FARMATODO	LOCATEL	FARMARKET	FARMACIAS SAAS	FARMAAHORRO	BADAN	FARMAPLUS	FARMAGO	BOTIQUERIA	FARMAVALOR	SUAF	RED VITAL	FARMACIA ACTUAL	TOTAL

**Amazonas**	5	2	3	1	2	1	2	0	1	1	1	1	0	20

**Anzoategui**	10	5	8	2	4	2	3	1	2	3	2	2	1	45

**Apure**	6	2	4	1	1	1	2	0	1	2	1	1	0	22

**Aragua**	12	6	9	3	5	3	4	1	2	4	3	3	1	56

**Barinas**	7	3	5	2	3	1	2	1	1	3	1	1	0	30

**Bolivar**	9	4	6	2	3	2	3	2	1	2	1	1	0	36

**Carabobo**	15	7	10	4	6	3	5	2	2	5	3	4	2	68

**Cojedes**	5	2	3	1	1	1	1	0	1	2	1	1	0	19

**Delta Amacuro**	4	1	2	1	1	0	1	0	0	1	1	1	0	13

**Falcon**	8	3	5	2	2	2	2	1	1	2	1	1	0	30

**Guarico**	4	2	3	1	1	1	1	0	1	1	1	1	0	17

**Lara**	11	5	8	3	4	2	3	1	2	4	2	2	1	48

**Merida**	8	4	6	2	3	2	2	1	1	3	2	1	1	36

**Miranda/Capital**	20	10	12	5	7	5	6	3	3	6	5	4	2	88

**Monagas**	7	3	5	2	2	2	2	1	1	2	1	1	0	29

**N Esparta**	6	2	4	1	2	1	2	1	1	2	1	1	1	25

**Portuguesa**	8	3	5	2	3	2	2	1	1	2	2	2	1	34

**Sucre**	6	3	4	1	2	1	2	1	1	2	1	1	1	26

**Tachira**	9	4	6	2	3	2	3	1	1	3	1	1	1	37

**Trujillo**	5	2	3	1	2	1	1	0	1	2	1	1	0	20

**Vargas**	4	2	3	1	1	1	1	0	1	1	1	1	1	18

**Yaracuy**	7	3	5	2	3	1	2	1	1	3	2	1	1	32

**Zulia**	18	8	12	4	7	4	5	2	2	5	3	3	1	74

**Total**	195	85	118	43	60	32	43	16	27	45	26	25	13	728

